# Genetic analysis in fetuses with isolated clubfoot: diagnostic insights and added value

**DOI:** 10.1038/s41431-025-01982-y

**Published:** 2025-11-21

**Authors:** Jana M. de Vries, Arda Arduç, Quinten Waisfisz, Maria B. Tan – Sindhunata, Brigitte HW Faas, Elisabeth van Leeuwen, Ingeborg H. Linskens, Eva Pajkrt

**Affiliations:** 1 Department of Obstetrics and Gynaecology, Amsterdam UMC, University of Amsterdam, Amsterdam, The Netherlands; 2 Amsterdam Reproduction and Development, Amsterdam, The Netherlands; 3 Department of Human Genetics, Amsterdam UMC, University of Amsterdam, Amsterdam, The Netherlands; 4 Department of Human Genetics, Radboud university medical center Nijmegen, Nijmegen, The Netherlands

**Keywords:** Skeleton, Genetics research, Genetic testing

## Abstract

This study evaluates the diagnostic genetic yield in fetuses sonographically suspect of having isolated clubfoot. We conducted a retrospective study on all fetuses with apparently isolated clubfoot on initial ultrasound, examined between January 2021 and December 2024. Clubfoot was classified as isolated when no additional structural anomalies were observed on initial imaging. Among 218 cases, 140 (64%) were classified as isolated. Prenatal genetic testing was performed in 64 of these cases (46%), of which 61 (95%) underwent both copy number variant (CNV) and single nucleotide variant (SNV) analysis. In 38 of the 61 (62%) cases targeted investigation of the *DMPK* gene was carried out too. Pathogenic or likely pathogenic causative variants were identified in six of the 61 (9.8%) pregnancies: two of the 26 tested (7.7%) with unilateral clubfoot and four of the 35 tested (11.4%) with bilateral clubfoot. These include SNVs in *TRPV4*, *PTPN11*, *BBS2*, and *MED13L* (4/61 = 6.6%) and two CNVs, a de novo 22q11.23 deletion, and a de novo 5q21.1q31.1 deletion (2/61 = 3.3%). One case remained unsolved due to the identification of a variant of uncertain significance (VUS) in *PIEZO2*. Three cases revealed unsolicited findings unrelated to the indication for testing. Our findings highlight the diagnostic yield of prenatal CNV- and SNV-testing in cases of suspected isolated clubfoot, but does not support systemic testing for *DMPK*. Although broad genetic testing can support diagnosis and counseling, challenges remain in interpreting results and managing unsolicited findings.

## Introduction

Congenital clubfoot (talipes equinovarus) is a spectrum with a complex deformity that affects the structure and alignment of the foot and ankle. It results in adduction, typically characterized by forefoot adduction, midfoot supination, hindfoot varus, and equinus positioning. Clubfoot has a prevalence of 1–3 per 1000 live births, and has a prenatal detection of 30–77% [1–3]. Clubfoot can be diagnosed during a fetal anomaly scan in the first and second trimester, and may present unilaterally or bilaterally, either as an isolated defect or in combination with other structural anomalies. In some cases, an underlying genetic disease is identified. Postnatal confirmation of clubfoot is required through physical examination. Its treatment typically follows the Ponseti method, involving serial casting, and in some cases surgical intervention [4].

Distinction between isolated and non-isolated clubfoot solely based on prenatal ultrasound examination can be challenging. Not all structural anomalies are visible at the time of examination, and some may only manifest later in pregnancy. Clubfoot can be an early symptom of syndromes involving the neuromuscular system, which may manifest later in pregnancy, such as arthrogryposis multiplex congenita (AMC) or neurodevelopmental disorders [4]. Prenatal invasive testing should be offered to enable parents to opt for assessing a potential underlying genetic condition [1]. A molecular genetic diagnosis helps to give future parents a tailored prognosis, thereby facilitating reproductive decisions and guiding perinatal and postnatal management.

Prenatal genetic testing for chromosomal and monogenic disorders can be performed using quantitative fluorescent-polymerase chain reaction (QF-PCR), chromosomal microarray analysis (CMA), and next generation sequencing (NGS)-based tests such as exome sequencing (ES), with the potential of analyzing Single Nucleotide Variants (SNVs) and Copy Number Variants (CNVs) from one set of data. The choice of test depends on local availability and protocols [1]. Myotonic dystrophy type I (OMIM #160900) is regarded to be in the differential diagnosis of prenatal clubfoot too. Therefore, analysis of the *DMPK* gene is frequently carried out, even though authors such as Leyne et al. and Dap et al. already suggested in their cohorts that the added value of testing for *DMPK* variants is low [5, 6].

The diagnostic genetic yield for CMA in isolated clubfoot is reported to be 2.2–11% [4–9]. In recent years, analysis of data generated by ES, being either SNV-analysis alone or SNV- and CNV-analysis simultaneously, has been implemented as a diagnostic tool for fetuses with any sort of structural anomalies on ultrasound [10–12]. For isolated clubfoot specifically, fewer than one hundred cases have been reported in literature in which ES-SNV analysis was conducted prenatally, with a diagnostic yield ranging from 4.3% to 25% [9, 13, 14]. Further research is urgently needed to explore the diagnostic yield of high resolution genetic analysis in unilateral and bilateral cases, since the studies of Huang et al., Pan et al. and Yu et al. [9, 13, 14] did not discriminate between these two categories. Therefore, the goal of this study is to explore the diagnostic yield of high resolution CNV- and SNV-analysis in case of apparently isolated clubfoot – in both unilateral and bilateral cases. We also took into account the yield of analysis of the *DMPK* gene.

## Methods

### Study design and participants

We performed a retrospective cohort study of all fetuses with prenatally isolated clubfoot diagnosed by ultrasound from January 2021 until December 2024 in the Fetal Medicine Unit (FMU) of Amsterdam UMC, a tertiary center. Cases were included when the initial targeted ultrasound examination – performed in the first or second trimester – resulted in a diagnosis of isolated clubfoot [15, 16]. A targeted ultrasound examination is a detailed and advanced fetal anomaly scan performed to assess fetal anatomy, after an anomaly is already suspected during a routine fetal anomaly scan. Clubfoot was considered isolated in case no other structural anomalies were found during this examination, with the exception of prenatal soft markers (e.g. plexus choroids cysts).

The sonographic diagnosis of clubfoot deformity was made when both long bones of the lower leg (tibia and fibula) were seen in the same plane as the sole of the foot throughout the entire examination. After the initial diagnosis of isolated clubfoot, parents were offered follow-up ultrasound examinations according to the care pathway that is carried out in the Amsterdam UMC in case of prenatally suspected contractures [17]. Typically, the second scan in our center is performed approximately two weeks after the initial ultrasound. The aim is to examine the fetal movement and screening for multiple contractures or other associated structural anomalies. This evaluation takes place before the upper limit for termination of pregnancy of 24 weeks, as regulated by the Dutch law [17, 18]. Additionally, a follow-up ultrasound is performed in the third trimester at 28–30 weeks of gestation to assess fetal growth, amniotic fluid levels, and signs of potential late development of neuromuscular symptoms (e.g. polyhydramnios, lunghypoplasia or micrognathia) [17]. Subsequently, all parents were offered prenatal counseling by a pediatric orthopedic surgeon to receive information on postnatal prognosis and treatment. Autopsy was offered in all cases of termination of pregnancy and was performed when parental consent was obtained. In addition, all cases underwent external physical examination. Children with isolated clubfoot are not systematically referred for follow-up genetic evaluation unless there are additional findings or developmental delay postnatally.

### Genetic testing

The testing took place either at the Amsterdam UMC or at the department of Human Genetics of the Radboud university medical center in Nijmegen, The Netherlands. Genetic analysis was performed on DNA isolated from either chorionic villi or amniotic fluid. In all cases, rapid aneuploidy testing by QF-PCR was first performed, to rule out the most commonly occurring aneuploidies, followed by CNV analysis by genomewide Chromosomal MicroArray (CMA), and in case of a normal CMA result by genepanel SNV analysis from ES data (ES-SNV), or by simultaneous exomewide CNV- and genepanel SNV-analysis from ES data (ES-CNV/SNV). For a comprehensive description of simultaneous CNV- and SNV-analysis from ES data, see reference [12]. CNV- and SNV-analysis for the fetus and the parents were preferably carried out simultaneously for optimal interpretation of the data. In addition, in part of the cases analysis of the *DMPK* gene was also requested by the clinical geneticist. Analysis of *SMN1* was not included in the exome sequencing pipeline used in this study and was not performed separately in any of the cases. However, targeted SMN1 testing can be requested prenatally.

The resolution of the genomewide CNV-analysis by CMA was 75 kb, whereas the resolution of the exomewide ES-CNV analysis was up to a single exon level. ES-SNV analysis was limited to genes in the requested gene panel(s), which were selected by the clinical geneticist. The most requested gene panels were panels including genes related to fetal akinesia, movement, skeletal, cognitive and/or Mendelian disorders.

All results were reported according to the guidelines of the American College of Medical Genetics and Genomics (ACMG) and only included pathogenic (P) and likely pathogenic (LP) variants [19]. The clinical significance of variants was systematically evaluated by referring to databases, scientific literature, and ultrasound findings, based on the human phenotype ontology [20].

The nomenclature of the detected variants was written in accordance with the International System for Cytogenomic Nomenclature (ISCN 2024) and the “Human Genome Variation Society” (HGVS) (https://varnomen.hgvs.org).

*DMPK* analysis was performed in accordance with the best practice guidelines, as previously published by Kamsteeg et al. [21].

### Statistical analysis

We characterized the data by using descriptive statistics, such as percentages and ratios, with differences assessed using chi-square. A significance level of *p* < 0.05 was applied for statistical significance. Statistical analyses were conducted using SPSS version 28 (IBM, Armonk, NY, USA). Categorical variables were compared using Fisher’s exact test.

Data regarding maternal characteristics, obstetrical history, sonographic findings, prenatal screening, invasive testing, pregnancy and neonatal outcomes were collected from patient records. We contacted the general practitioners and parents by telephone to obtain missing information from hospital records. Cases where medical records or telephone questionnaires were not available were marked as lost to follow-up. This study was approved by the Medical Ethics Committee of Amsterdam UMC (W21_361 # 21.401) regarding general follow-up data on our FMU.

## Results

A total of 218 cases of clubfoot were identified in our FMU between January 2021 and December 2024. Of these, 140 cases (64%) were isolated at the initial targeted anomaly scan and thus included in this study. Of these 140, 63 (45%) were diagnosed with unilateral clubfoot, and 77 (55%) with bilateral clubfoot. Pregnancy was terminated in 11 cases (8%). In total, parents opted for invasive prenatal testing in 64 of the 140 cases (46%). The characteristics of the 64 tested cases concerning the maternal age, family history of clubfoot, performed noninvasive prenatal testing (NIPT), gestational age at initial targeted anomaly scan, and pregnancy outcome are shown in Table 1.

**Table 1:** Baseline characteristics.

Maternal characteristics (*n* = 64)	Number (%)
Mean maternal age at diagnosis in years ±range	33.1 ± 4.4
Family history of clubfoot	5 (7.8%)
NIPT test performed	55 (85.9%)
Gestational age at diagnosis	17 + 5 ± 3 + 4
Pregnancy outcome	
Live born	54 (84.4%)
Termination of pregnancy	9 (14.1%)
Neonatal death	1 (1.5%)

In three cases, SNV-ES analysis was not performed because QF-PCR and CMA revealed no abnormalities, and the parents chose not to proceed with further testing. Finally, in 61 of the 64 (95%) cases ES was opted – all with a normal QF-PCR result.

The abnormal findings of the SNV- and CNV-analyses are listed in Table 2a. A pathogenic or likely pathogenic causative variant was identified in 6 of the 61 cases (9.8%): in 2 of the 26 unilateral cases (7.7%) and in 4 of the 35 bilateral cases (11.4%). This difference was not statistically significant (*p* = 0.69). The abnormal SNV findings include SNVs in *TRPV4*, *PTPN11*, *BBS2*, and *MED13L* (4/61 = 6.6%). The father of case 1 had muscle weakness of the shoulder girdle and lower limbs, which following the prenatal diagnosis, is consistent with the diagnosis of TRPV4-related scapuloperoneal spinal muscular atrophy (SPSMA). In addition to the four SNVs, there were causative CNVs identified in two cases with bilateral clubfoot, a de novo 22q11 deletion, and a de novo 5q21.1q31.1 deletion (2/61 = 3.3%), the latter having been identified by NIPT, previously performed in the first trimester. In the case with the 22q11.2 deletion, the father’s cousin also had clubfeet; however, no genetic testing was performed in that relative, and it was known that this was a de novo deletion.

**Table 2:** a. Findings of ES-SNV (CMA negative) or ES-CNV/SNV analysis in 61 women who opted for invasive prenatal testing and ES; b. Unsolicited findings, unrelated to the indication for testing, identified by ES-SNV analysis in 61 women who opted for invasive prenatal testing and ES.

Case	laterality	GA	NIPT	Inheritance	Variant: Nucleotide alteration, deduced protein change	ACMG criteria	Aggregated pathogenicity prediction	MAF	Pregnancy outcome	Pre-/postnatal findings
**a**										
**1**	uni	18 + 2	Yes	AD	*TRPV4*: Chr12(GRCh37):g.110252312G>C NM_021625.5:c.290C>G p.(Pro97Arg) (paternal)	LP: PS3, PM2	Uncertain (0.6)	–	A, 41 + 1	Bilateral clubfeet, follow-up at 2 yrs: mild proximal and distal muscle weakness.
**2**	bi	20 + 2	No	DN	*PTPN11*: Chr12(GRCh37):g.112888150A>G NM_002834.5:c.166A>G p.(Ile56Val)	P: PS4, PS2, PM1, PM2, PM5, PP2, PP3, PP5	Deleterious (0.74)	0.000001240	TOP, 23 + 4	No additional findings
**3**	uni	20 + 3	Yes	AR (compound heterozygous)	*BBS2*: Chr16(GRCh37):g.56540122_56540123del NM_031885.5:c.627_628del p.(Cys210Serfs*20) (maternal) *BBS2*: Chr16(GRCh37):g.56532462G>A NM_031885.5:c.1546C>T p.(Gln516*) (paternal)	P: PVS1, PM2, PM3, PM5 P: PVS1, PM2, PM3, PM5	– –	0.000001859 –	TOP, 23 + 6	Postaxial polydactyly both hands, unilateral clubfoot
**4**	bi	20 + 4	Yes	DN	*MED13L*: Chr12(GRCh37):g.116401310_116401336inv NM_015335.5:c.6388-12_6402inv r.spl	LP: PVS1, PS2, PM2	–	–	TOP, 23 + 4	Down slanting palpebral fissures and hypertelorism. Notably low-set and posteriorly rotated ears. Curved nasal tip. Small chin and intact palate. Hands: slender, long fingers Feet: pronounced bilateral pes equinovarus. Camptodactyly of toes 3 to 5.
**5**	bi	19 + 6	Yes	DN	Seq[GRCh37] del(22)(q11.21 q11.21) Chr22:g.(18659584_18893887)_(21414817_21416012)del	P			TOP, 23 + 2	No additional findings
**6**	bi	14 + 3	Yes, abnormal: del(5)(q21.1q31.1)	DN	seq[GRCh37] 5q21.1q31.1(100147550_131411545)x1 NC_000005.9:g.(98262090_100147550)_(131411545_131528702)del	P			TOP, 19 + 6	No additional findings
**7**	bi	19 + 3	Yes	AR (Compound heterozygous)	*PIEZO2*: Chr18(GRCh37):g.10857132G>A NM_022068.4:c.570C>T p.(Gly190=) / r.spl? (paternal); *PIEZO2*: Chr18(GRCh37):g.10871249A>G NM_022068.4:c.492+2T>C p.? (maternal)	VUS: PM2 LP: PVS1, PM2	Deleterious (0.79) SpliceAI: Splice-Altering / strong (0.97) Deleterious (0.8) SpliceAI: Splice-Altering / strong (0.99)	6.503e−7 –	TOP, 22 + 5	Abnormal quality of the fetal movement. Postnatally also contractures wrists, elbows, knees and fingers.
**b**										
**8**	uni	19 + 4	Yes	AD (heterozygous)	*BRCA2*: Chr13(GRCh37):g.32906762del NM_000059.3:c.1147del p.(Ile383fs) (paternal)	P: PVS1, PS4		0.000001859	A, 40 + 2	No clubfoot
**9**	uni	19 + 4	Yes	AR	*FLG*: Chr1(GRCh37):g.152285861G>A NM_002016.1:c.1501C>T p.(Arg501*) (maternal) *FLG*: Chr1(GRCh37):g.152277415G>C NM_002016.1:c.9947C>G p.(Ser3316*) (paternal)	LP: PVS1, PM2, PP5 LP: PVS1, PM2, PP5		0.01696 0.0004096	TOP, 23 + 6	No additional anomalies. Posteromedial bowing suspected.
**10**	bi	21 + 3	No	AR (homozygous)	*SERPINC1*: Chr1(GRCh37):g.173883708G>A NM_000488.4:c.391C>T p.(Leu131Phe) (both parents)	P: PS4, PM2, PP1, PP2, PP3	Deleterious (0.86)	0.00001115	A,41 + 3	Continued: not causal for clubfoot. Postnatally no clubfoot, but mild inversion of the foot

Classification following ACMG Criteria 2015.

*uni* unilateral, *bi* bilateral, *GA* gestational age, *wks* weeks, *TOP* termination of pregnancy, *A* alive, *DN* de novo, *AR* autosomal recessive, *AD* autosomal dominant, *MAF* Minor Allele Frequency according to gnomAD v4.1.0, *NIPT* non-invasive prenatal testing.

ES-SNV analysis also revealed compound heterozygous variants in *PIEZO2* in case 7, of which the c.492+2T>C variant was likely pathogenic, and the c.570C>T variant was considered a variant of uncertain significance (VUS) (Table 2a). In three cases (3/61 = 4.9%), ES-SNV analysis revealed unsolicited findings unrelated to the indication for testing. One fetus and its mother carried a pathogenic variant in *BRCA2*; another fetus appeared compound heterozygous for two pathogenic variants in *FLG*; and a third fetus appeared homozygous for a pathogenic variant in *SERPINC1* (Table 2b).

In 38 of the 61 cases (62%), testing for myotonic dystrophy type 1, caused by a (CTG)n repeat expansion in the 3’UTR of the *DMPK* gene, was additionally performed, and none carried the expansion.

Postnatally, 5 of the 76 children (5.3%) with isolated clubfoot who had not undergone prior genetic testing received genetic evaluation, resulting in the diagnosis of trisomy 21 in one case. The other four were tested due to: (1) intrauterine fetal death at 39 weeks, (2) prematurity with pulmonary, thoracic, renal, and limb anomalies, (3) termination after abnormal fetal movements with clubfeet, and (4) paternal carrier status of a microduplication. The remaining 71 children were all clinically evaluated and did not show any signs of other underlying pathology.

## Discussion

In this study we investigated the diagnostic genetic yield in fetuses with isolated clubfoot, providing valuable insights into the genetic etiology of this condition. Among the evaluated cases, we identified causative CNVs or SNVs in six cases (6/61 = 9.8%), four of which were SNVs (4/61 = 6.6%) and two CNVs (2/61 = 3.3%).

The variants in *TRPV4, PTPN11, BBS2*, and *MED13L* were all classified as pathogenic or likely pathogenic and causative for clubfoot. *TRPV4* variants are associated with a broad spectrum of neuromuscular disorders with clinical phenotypes that can vary even within the same family carrying identical variants [22, 23]. Variants in *PTPN11* are associated with Noonan syndrome [24–26], with clubfoot as one of the (rare) features. In addition, while a clubfoot is observed in only 1.8% of cases with Bardet-Biedl syndrome (BBS), renal anomalies (85%) and polydactyly (71%) are the most frequent findings [27, 28]. In the case with pathogenic variants in *BBS2*, the diagnosis of bilateral postaxial polydactyly had not been made prenatally. Although polydactyly is generally considered a detectable anomaly during second-trimester scans, its visualization can be hampered by various factors, such as maternal obesity, suboptimal fetal position, or acoustic shadowing from uterine anomalies, particularly when the polydactyly lacks a bony content [29]. Moreover, in the absence of other anomalies, subtle digital findings may be easily overlooked [29]. This highlights the importance of systematic assessment of the hands and feet during prenatal imaging and the need for awareness that even seemingly isolated limb anomalies may have an underlying syndromic or genetic etiology. Pathogenic heterozygous variants in *MED13L* are associated with an autosomal dominant syndrome characterized by intellectual disability and distinct facial features, with or without congenital heart defects (OMIM #616789) [30, 31]. Clubfoot has also been reported for patients with this condition [32].

Case 5 showed a de novo 22q11 deletion, that is linked to 22q11.2 deletion syndrome (OMIM #611867), a well-known pathogenic condition associated with clubfoot [5]. Case 6 presented with a pathogenic de novo deletion in chromosome band 5q21.1q31, known to be associated with clubfoot and developmental disorders [33–36]. This large deletion of 31–33 Mb includes the *FBN2* gene, which is linked to arachnodactyly, joint contractures, kyphoscoliosis, and variable systemic involvement (OMIM #121050) [33–36]. The extent of neurodevelopmental impairment could not be precisely predicted, but substantial impairment was considered likely based on the deletion size and gene content.

Case 7 remained unresolved due to a VUS. *PIEZO2* is associated with distal arthrogryposis with impaired proprioception and touch (DAIPT), and the fetus’ phenotype was consistent with this condition, including the abnormal quality of the fetal movements [37, 38]. Although these findings suggest the *PIEZO2* variants are likely disease-causing, the c.570C>T variant remains classified as a VUS in the absence of RNA analysis confirming its predicted effect on splicing. This variant is predicted to be synonymous at the protein level while splice prediction tools suggests that this variant may result in a cryptic splice donor site in exon 6 (NM_022068.4). This case illustrates how postnatal follow-up can provide valuable insights into variant interpretation and supports the need for longitudinal phenotyping when initial classifications remain uncertain. These findings may contribute to future reclassification of the variant as likely pathogenic or pathogenic.

The diagnostic SNV-yield has been evaluated by three previous reports. Yu et al. analyzed data of 38 singleton pregnancies diagnosed with isolated clubfoot [14]. Trio-based SNV-exome sequencing analysis was conducted after a normal CMA result. Pathogenic or likely pathogenic variants were identified in 4 of the 38 cases (10.5%), which is slightly higher than our finding of 6.6%. The genes involved in the study of Yu et al. were *BRPF1*, *ANKRD17*, *FLNA*, and *KIF1A* [14]. These genes are associated with musculoskeletal and neurodevelopmental syndromes. Another retrospective study by Huang et al., with 83 singleton pregnancies diagnosed with clubfoot (both isolated and non-isolated), identified clinically significant SNVs in 12% of cases, with a higher detection rate in non-isolated clubfoot (22.2%) compared to isolated cases (4.3%) [13]. This latter finding being slightly lower than ours. Pathogenic SNVs were found in two of the 47 fetuses with isolated clubfoot, one in the *TGM6* gene and another in the *BRPF1* gene [13]. In 2025, Pan et al. showed an SNV yield of 25% by ES data analysis in isolated clubfoot in a group of four fetuses with isolated clubfoot [9]. This high percentage is likely due to the low number of tested fetuses with an isolated clubfoot [9]. Together with our study, these three studies show that apparently isolated clubfoot can have an underlying genetic cause, highlighting the importance of parental counseling.

Genetic testing not only increases the likelihood of identifying an underlying genetic cause, but also the likelihood of identifying unsolicited findings. In the current study, we identified causative variants in 9.8% (*n* = 6), and unsolicited findings, unrelated to the indication for testing in 4.9% of the fetuses (*n* = 3). Depending on the type of unsolicited findings (fetal, parental or both, mild or severe phenotype), such findings can present ethical dilemmas and psychological burdens for parents, highlighting the importance of comprehensive pre-test counseling to discuss possible outcomes and their implications [39].

As in none of the cases with *DMPK* analysis a causative repeat length was detected, the data of our cohort do not support routine *DMPK* analysis as part of the genetic work-up in isolated clubfoot, which is in accordance with previous reports [5, 6, 21].

A prenatal ultrasound diagnosis of isolated clubfoot should be handled with caution, as it may not always indicate an entirely isolated clubfoot [37]. When using prenatal ultrasound techniques to detect fetal abnormalities, several limitations must be considered. Firstly, the sensitivity of ultrasound techniques is dependent on various factors, including fetal size and positioning, maternal body type, the resolution of ultrasound equipment, and the experience of the sonographer [40]. Secondly, not all abnormalities are visible on ultrasound, especially those that do not manifest as clear structural or functional anomalies (e.g. intellectual disability or subtle neurodevelopmental disorders), leading to potential missed diagnoses [41–43]. These conditions may only become apparent years after birth, often around school age, when developmental milestones and cognitive performance can be more accurately assessed [43]. As the current follow-up period is limited, it is possible that additional cases in the genetically untested group may eventually show signs of associated abnormalities that were not evident in the neonatal period. To date, only five cases underwent postnatal genetic testing, with trisomy 21 identified in one of them. However, since postnatal genetic testing was not systematically performed in our cohort, no definitive conclusions can be drawn regarding the overall genetic yield. These findings underscore the importance of long-term follow-up and suggest that genetic abnormalities may be underestimated in cases with clubfoot initially classified as isolated.

Notably, among the five cases with a positive family history of clubfoot, one (20%) had a pathogenic variant identified, compared to 5 out of 59 cases (8.5%) without a family history. While this observation may indicate a potentially higher diagnostic yield in the presence of family history, causality was excluded in this case as the 22q11.2 deletion occurred de novo. In particular, isolated clubfoot often reflects multifactorial inheritance, and the presence of clubfoot in a relative does not necessarily imply a monogenic cause. These nuances should be clearly communicated during counseling and considered when deciding on genetic testing.

All clubfoot in our cohort were confirmed during targeted ultrasound examinations after referral because of an abnormal routine scan. Among the group with pathogenic or likely pathogenic variants, additional anomalies were observed in two cases, and only during post-mortem examination. Postnatal examinations after termination of pregnancies showed multiple contractures in the case with variants in *PIEZO2* - potentially due to the time interval between the last ultrasound and termination, as isolated contractures can progress to AMC – and polydactyly of both hands in the case with variants in *BBS2*. This highlights the added value of genetic analysis in cases of isolated clubfoot, as, in the majority of our cohort, clubfoot was the only detectable phenotype of the underlying genetic disorder during prenatal ultrasound examination. The strengths of this study include its focus on a well-defined cohort of cases with prenatally diagnosed isolated clubfoot, the detailed and systematic genetic testing, and the ability to differentiate between unilateral and bilateral cases. The study contributes to the limited existing data on comprehensive SNV analysis in isolated clubfoot cases. Although the number of cases remains limited, this study represents the largest prenatal cohort reported to date. The results support that, in cases of isolated clubfoot, prenatal genetic testing should include chromosomal, CNV- and SNV-analysis, using high-resolution genetic techniques.

In conclusion, our study demonstrates that prenatal CNV- and SNV-analysis yields a molecular diagnosis in approximately 10% of fetuses with apparently isolated clubfoot, supporting its diagnostic value even in the absence of other structural anomalies. Our findings show that both unilateral and bilateral cases may harbor causal pathogenic or likely pathogenic variants. This underscores the importance of offering genomic testing regardless of laterality. At the same time, challenges remain regarding the likelihood and interpretation of uncertain or unsolicited findings, and careful pre- and postnatal counseling is essential to ensure that testing contributes meaningfully to the parental decision.

## Summary

### What’s already known about this topic?

- Prenatal ultrasound examination only cannot reliably distinguish between isolated and non-isolated clubfoot.
- Prenatal invasive testing should be offered to assess potential underlying genetic conditions in case of fetal clubfoot.
- For isolated clubfoot specifically, fewer than one hundred cases have been reported in which SNV analysis from exome data was conducted prenatally, with a diagnostic yield ranging from 4.3% to 25%.

### What does this study add?

- This study presents the largest prenatal cohort to date with DMPK-, CNV- and SNV-analysis in fetuses with sonographically isolated clubfoot.
- Bilateral isolated clubfoot does not appear to have a higher likelihood of being caused by a genetic condition compared to unilateral isolated clubfoot.
- The study highlights the importance of genetic counseling – as broad genetic analysis might not only detect causative variants, but both future parents and healthcare providers may be confronted with challenges such as unsolicited or unresolved findings.

## Data Availability

Datasets generated and/or analyzed during the current study are available from the corresponding author on reasonable request.

## Code availability

Datasets generated and/or analyzed during the current study are available from the corresponding author on reasonable request.
